# Network analysis of Down syndrome and SARS-CoV-2 identifies risk and protective factors for COVID-19

**DOI:** 10.1038/s41598-021-81451-w

**Published:** 2021-01-21

**Authors:** Ilario De Toma, Mara Dierssen

**Affiliations:** 1 Centre for Genomic Regulation (CRG), The Barcelona Institute of Science and Technology, Barcelona, Spain; 2 Universitat Pompeu Fabra (UPF), Barcelona, Spain; 3 Biomedical Research Networking Center On Rare Diseases (CIBERER), Institute of Health Carlos III, Madrid, Spain

**Keywords:** Gene regulatory networks, SARS-CoV-2

## Abstract

SARS-CoV-2 infection has spread uncontrollably worldwide while it remains unknown how vulnerable populations, such as Down syndrome (DS) individuals are affected by the COVID-19 pandemic. Individuals with DS have more risk of infections with respiratory complications and present signs of auto-inflammation. They also present with multiple comorbidities that are associated with poorer COVID-19 prognosis in the general population. All this might place DS individuals at higher risk of SARS-CoV-2 infection or poorer clinical outcomes. In order to get insight into the interplay between DS genes and SARS-cov2 infection and pathogenesis we identified the genes associated with the molecular pathways involved in COVID-19 and the host proteins interacting with viral proteins from SARS-CoV-2. We then analyzed the overlaps of these genes with HSA21 genes, HSA21 interactors and other genes consistently differentially expressed in DS (using public transcriptomic datasets) and created a DS-SARS-CoV-2 network. We detected COVID-19 protective and risk factors among HSA21 genes and interactors and/or DS deregulated genes that might affect the susceptibility of individuals with DS both at the infection stage and in the progression to acute respiratory distress syndrome. Our analysis suggests that at the infection stage DS individuals might be more susceptible to infection due to triplication of *TMPRSS2*, that primes the viral S protein for entry in the host cells. However, as the anti-viral interferon I signaling is also upregulated in DS, this might increase the initial anti-viral response, inhibiting viral genome release, viral replication and viral assembly. In the second pro-inflammatory immunopathogenic phase of the infection, the prognosis for DS patients might worsen due to upregulation of inflammatory genes that might favor the typical cytokine storm of COVID-19. We also detected strong downregulation of the *NLRP3* gene, critical for maintenance of homeostasis against pathogenic infections, possibly leading to bacterial infection complications.

The recent outbreak of the novel severe acute respiratory syndrome SARS-coronavirus 2 (SARS-CoV-2) has caused more than 86 million cases of COVID-19 and is responsible for almost 2 million deaths (08/01/2020; https://covid19.who.int/), with figures expected to rise in the coming months. COVID-19 is particularly severe in elderly individuals (> 60 years old), especially those with chronic medical conditions (i.e., hypertension, diabetes mellitus, cardiovascular disease, chronic respiratory disease or cancer). However, fast spread of SARS-Cov-2 infection has precluded the study of other possibly vulnerable populations, such as individuals with intellectual disability (ID). Down syndrome (DS) is the most common genetic form of ID. Individuals with DS present with comorbidities, such as obesity, type I diabetes or congenital heart disease (CHD), that are associated with poorer COVID-19 prognosis in the general population. Hypotonia, developmental delay, obstructive sleep apnea, craniofacial anomalies, immune deficiency, and cardiac problems, as well as gastroesophageal reflux, are also frequent in DS and contribute to the increased risk of respiratory tract infections^1^. Individuals with DS also develop more severe complications during other viral respiratory infections such as influenza^2^ and respiratory syncytial virus (RSV)^3^, including pneumonia, hospitalization, intubation and greater mortality due to secondary bacterial infections. Another risk factor is that immune response is substantially impaired in DS^4^. Deficits in trisomic individuals include functional anomalies in a variety of immune cells (i.e., T- and B-cells, monocytes and neutrophils), and, of importance to vaccine-development, suboptimal antibody responses^5^. A number of studies found reductions in T and B cells, and increases in serum inflammatory cytokine levels in peripheral blood of people with DS, suggesting suboptimal immunization^6^. In the general population, severe COVID-19 patients typically show increased cytokine levels (IL-6, IL-10, IL-2, IL-7 and TNF-α)^7^, lymphopenia (in CD4+ and CD8+ T cells), and decreased IFN-γ expression in CD4+ T cells. Those are associated with severe COVID-19 and the so-called “cytokine storm”^8^, an excessive immune response to external stimuli that predicts worse COVID-19 prognosis^9^ and correlates with the severity of pathogenic coronavirus infections^10^. DS is characterized by an ‘inflammatory priming’ of immune cells, which is in line with the elevated levels of cytokines^11^ in blood in the absence of viral infections^12^. Furthermore, the interferon response (IFN), key for initiating and amplifying cytokine release, is chronically overactive in DS^13^ and Interferon Alpha and Beta Receptor Subunits 1 and 2 (IFNAR1 and IFNAR2), that form a heterodimeric interferon receptor, are also triplicated in trisomic cells. Finally, recent evidence suggests that an increase in Transmembrane protease serine 2 (TMPRSS2) expression results in increased infection/establishment of infection with lower viral titers. Cell entry of coronaviruses depends on binding of the viral spike (S) proteins to cellular receptors and on S protein priming by host cell proteases. The *TMPRSS2* gene, located on Chr21q22^14,15^, encodes for a serine protease that proteolytically activates the S protein for its interaction with the angiotensin-converting enzyme 2 (ACE2) as the entry receptor, allowing the entrance of the SARS-CoV-2 virus in the host cell^16^. In fact, a TMPRSS2 inhibitor approved for clinical use blocked entry has been proposed as a treatment option^15^.

In theory, all these factors could lead to a more vulnerable scenario in DS, induced by the triplication of the genes encoded by human chromosome 21 (HSA21). However, gene expression studies showed that, even if HSA21 shows the highest percentage of differentially expressed genes, the transcriptome deregulation extends genome wide, with many non-HSA21 genes upregulated or downregulated^17,18^. To understand the potential differential impact of COVID19 in individuals with DS compared to the general population, we here mapped the transcriptomic changes induced by the trisomy onto the pathways and proteins known to be affected by SARS-CoV-2. We reasoned that besides *TMPRSS2*, other genes consistently deregulated in DS could define genetic risk factors of DS-COVID19 comorbidity. We analyzed 69 DS transcriptomic and proteomic studies and we detected that genes mapping on HSA21 were consistently deregulated across different tissues and states. We also reasoned that the levels of proteins interacting with or regulated by HSA21 genes (HSA21 interactors) are more likely to be deregulated in cases of trisomy 21. We then analyzed their overlap with molecular pathways involved in COVID-19 and the host proteins interacting with viral proteins from SARS-CoV-2 and created a DS-SARS-CoV-2 network.

## Methods

### Sources

In order to get insight on the interplay between DS genes and SARS-cov2 infection and pathogenesis we looked at the pathways related with infection with corona-virus as reported in *WikiPathways* (https://www.wikipathways.org/index.php/Portal:Disease/COVIDPathways) and at the recently published SARS-CoV-2-Human Protein–Protein Interactome^19^. Protein–protein interactions were retrieved from the STRING database version 10, using a score cut-off ≥ 215 for the network, and > 900 for the HSA21 interactors.

Transcriptomic data were extracted from Gene Omnibus Expression GEO, and Array Express. Supplementary Table 1 lists all datasets that were used in this analysis, with their references.

### Analysis of micro-array data

For the analysis micro-array data–quality check, background correction, and normalization–we used two different R packages, because of the different platforms used.

- “Affy”^20^ and “oligo”^21^ for the analysis of Affymetrix GeneChip data at the probe level
- “Beadarray”^22^ for illumina bead-based arrays

In most cases the differential expression analysis of microarray data was performed using moderated t-statistics with the package limma^23,24^. When subsetting the samples in different contrasts was not possible due to the sample size, we used the function *duplicateCorrelation* function from the *statmod* package^25^, for blocking for variables other than the trisomy (e.g. sex or age).

### Analysis of RNA-seq data

All RNA-sequencing experiments were from illumina. In this case, reads were downloaded from the *Sequence Read Archive* using fastq-dump and mapped to the *mm10* for mouse and *GRCh38.p12* for human samples. For gene annotation of the mapped reads, we used *gencode.vM17* for mouse and *gencode.v28* for human samples. Differential expression analysis between trisomic and wild type samples was performed with *DESeq2*^26^, blocking for factors other than the trisomy (sex, age, etc.) when possible.

### Gene annotation

For the annotation of the ensembl gene identifiers (IDs) we used the *biomaRt* package, using the *getLDS* function to map mouse ensembl gene IDs to their human orthologous; the org.Mm.eg.db and org.Hs.eg.db packages^27,28^.

For the annotation of the microarray probes we used the following R packages:

- Affymetrix Human Genome U133 Set: hgu133a.db
- Affymetrix Human Genome U133 Plus 2.0 Array: hgu133plus2
- Affymetrix Murine Genome U74v2: mgu74av2.db
- Affymetrix huex10 annotation data: huex10sttranscriptcluster.db
- Affymetrix hugene10 annotation data: hugene10sttranscriptcluster.db
- Affymetrix hugene20 annotation data: hugene20sttranscriptcluster.db
- Affymetrix mogene10 annotation data: mogene10sttranscriptcluster.db
- Affymetrix Mouse Genome 430 2.0 Array annotation data: mouse4302.db
- Illumina HumanWG6v2 annotation data: illuminaHumanv2.db
- Illumina MouseWG6v2 annotation data: illuminaMousev2.db
- Illumina HumanHT12v4 annotation data: illuminaHumanv4.db
- Illumina HumanHT12v3 annotation data: illuminaHumanv3.db

### Detection of consistently differentially expressed genes

We selected as differentially expressed **(**DE) the top 500 genes changing at least 1.5 times with a Benjamini adjusted p-value < 0.05 in at least one trisomic vs. euploid comparison. However, in order to define “consistently DE” genes, we analyzed the heavy tail distribution of differential expression across all DE genes and selected genes DE at least 4 times, which corresponded to less than 5% of the distribution. Graph-based analysis and visualization was performed with the igraph package.

## Results

**Figure 1:**
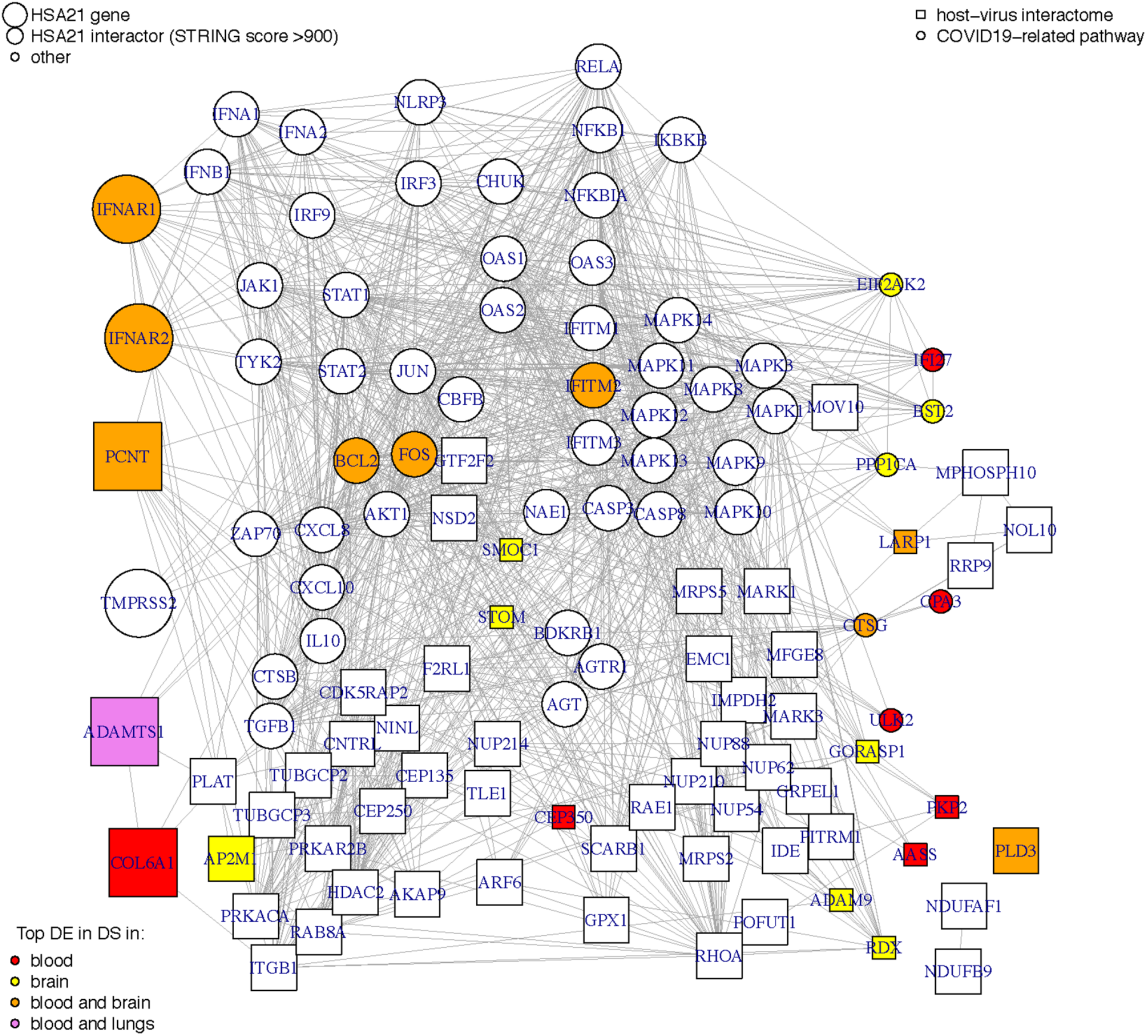
DS-SARS-CoV-2 network. Molecular interaction network where the size of each node represents a HSA21 gene (bigger size), a HSA21 interactor (medium size, STRING score ≥ 900) or a non-HSA21 gene (smaller size) found consistently deregulated in DS. Circles represent the genes related to infection with SARS-CoV-2 as reported in *WikiPathways*, and squares are host proteins found in the SARS-CoV-2-Human Protein–Protein Interactome. Genes found consistently DE in at least 4 DS transcriptomic studies are colored depending on the tissue: in red when mainly DE in blood, yellow when mainly deregulated in brain, and orange when found in both blood and brain tissue. The gene* ADAMTS1* is deregulated in blood but is also a pro-angiogenic factor upregulated in DS lungs (in purple)^29^. The edges indicate a protein–protein interaction score (STRING score ≥ 215).

To get insight into the interplay between genes consistently deregulated in DS and SARS-cov2 infection and pathogenesis we built a DS-SARS-CoV-2 network. We labeled as “DS genes” three categories of genes: HSA21 genes, HSA21 interactors, and non-HSA21 genes found consistently deregulated in at least 4 DS transcriptomic studies (Supplementary Table 1). We then mapped these DS genes onto COVID-19 related pathways (as reported in WikiPathways) and on the interactome^19^ coming from a mass-spectrometry affinity study with proteins of SARS-CoV-2^19,^ ending up with 114 DS genes that could determine a differential susceptibility to COVID-19 infection. We therefore connected these genes based on their functional and physical annotated interactions (Fig. 1, Supplementary Fig. 1).

Approximately half of the nodes in the DS-SARS-CoV-2 network (55/114 nodes) contained proteins involved in host Covid-19 related pathways, while the other half (59/114 nodes) contained host proteins known to interact with viral proteins. Six of these nodes were HSA21 genes, and 92 HSA21 interactors. Overall, 26 genes were differentially expressed in at least 4 DS transcriptomic studies, 16 of which were neither a HSA21 gene nor a HSA21 interactor (Fig. 1).

**Figure 2:**
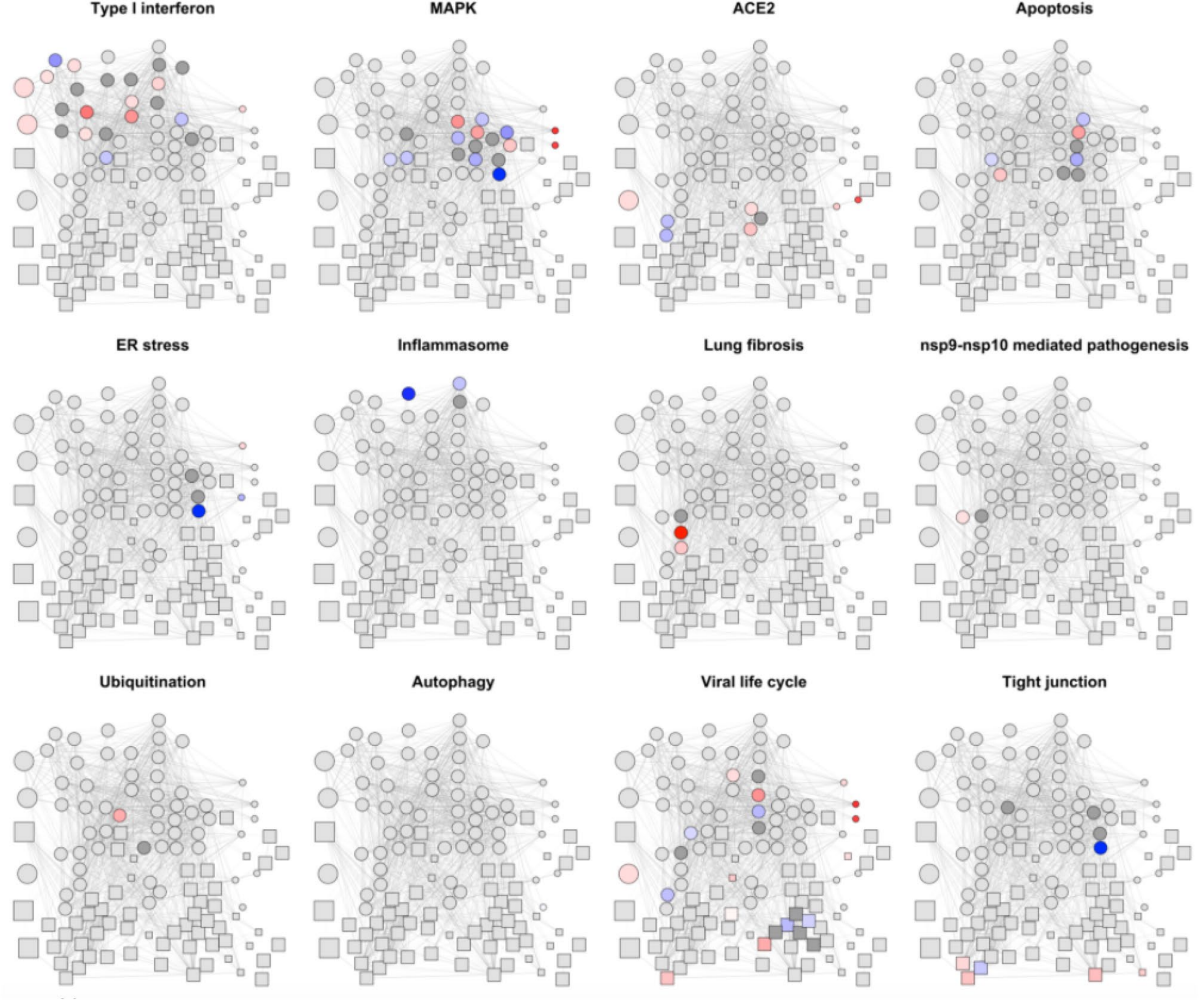
The same network as in Fig. 1 is shown here for each of the *WikiPathways*, the biological process “Viral life cycle” and the KEGG pathway tight junction^30^. The color code indicates the gene mean log2FC from genes upregulated in DS (red) to genes downregulated in DS (blue). When this information is not known the gene is in dark gray color.

In this network we detected several pathway connected with COVID-19 that are affected in DS that will be discussed in the following sections (Fig. 2).

### Mechanisms of viral entry

#### Trisomy of *TMPRSS2* might enhance virus activation

**Figure 3:**
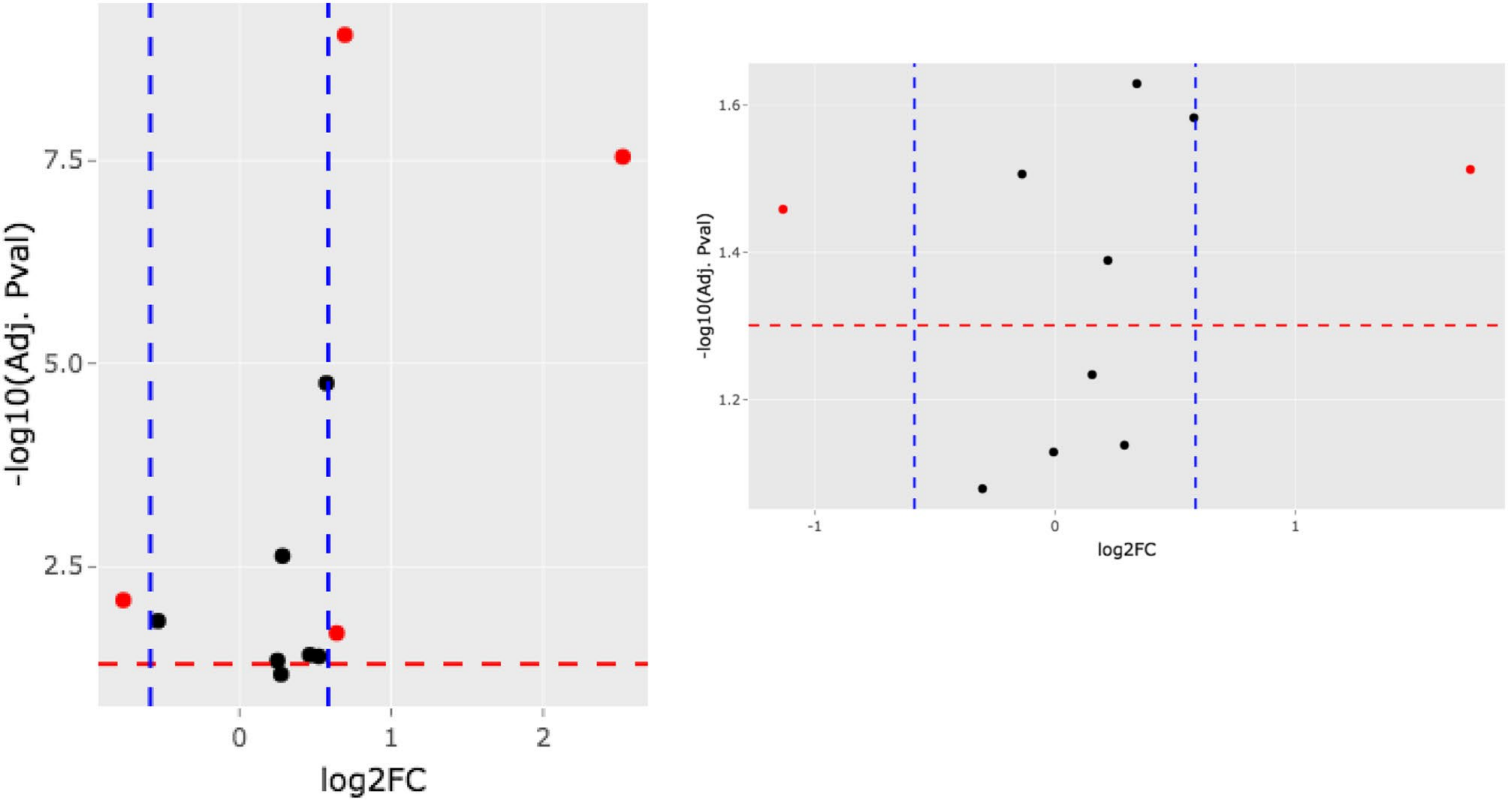
Volcano plot of the *TMPRSS2* and *ACE2* genes. (**A**) Volcano plot showing on the x-axis the log2 fold change and the y-axis the significance (− log10 of the Benjamini-corrected p-value) across different DS transcriptomic studies detecting *TMPRSS2*. Red dots indicate contrasts with deregulation > 1.5 of absolute fold change (dashed blue lines) and < 0.05 adjusted p-value (dashed red line). (**B**) Same as in (**A**) but for *ACE2*.

*TMPRSS2* was upregulated across the different DS studies, with a median fold change of 1.59, in cortical tissue^31,32^, white blood cells^33^, lymphoblastoid cell lines^31^, iPSCs^34^, mouse fetal liver and placental tissue^35^ (Fig. 3A). This suggests that the proteolytic activation of the viral spike protein (S-protein) for its interaction with the receptor ACE2, would be favored in DS, allowing increased entrance of the SARS-CoV-2 virus in the host cell. Conversely, we found downregulation of the endosomal protease cathepsin B (CatB) that, similarly to cathepsin L (CatL), could also prime S-protein, suggesting that the preferential virus entry in DS is through TMPRSS2. In fact, only TMPRSS2 activity is essential for viral spread and pathogenesis in the infected host whereas CatB/L activity is dispensable^36–38^. However, *ACE2* itself is not consistently differentially expressed, and it is not even a HSA21 interactor. We found *ACE2* (Fig. 3B) upregulated in DS human induced pluripotent stem cells (iPSCs) ^34^, downregulated in peripheral blood from DS individuals^39^; slightly upregulated in the hippocampus of the DS model Ts1Cje (6–7 months old)^40^, DS human postnatal inferior temporal cortex^32^ and adult dorsolateral prefrontal cortex^32^, and slightly downregulated in post-mortem dorsolateral DS prefrontal cortex^41^.

### Other *ACE-2* related mechanisms

Interestingly, in DS we detected an upregulation of the bradykinin receptor B1 (BDKRB1)^32,42^, one of the HSA21 interactors revealed in our analysis, and of the metalloprotease CPA3, that is normally upregulated in asthma patients. *BDKRB1* upregulation, as part of the kallikrein-kinin system, could determine a higher susceptibility in DS individuals to ARDS. Following viral binding, *ACE2* expression and activity is eventually downregulated by the virus through multiple mechanisms, preventing it from performing its usual function in states of health^43^. The downregulation of *ACE2* signaling induces the kallikrein-kinin system which is activated during inflammatory conditions with vascular-alveolar fluid extravasation, leukocyte extravasation and recruitment to the lung and acute respiratory distress syndrome (ARDS), lung injury and pneumonia^44^.

### The interferon signaling is activated in DS

We detected 21 genes in our network belonging to the IFN-I signalling pathway, and several of them were found upregulated in DS transcriptomic datasets: *IFNAR1*^13,32,45,46^*, IFNAR2*^13,32,35,45,47–50^*, IFNA2*^32^*, IFNB1*^32^*, STAT1*^45^*, STAT2*^51^*, OAS1*^13,52^*, OAS2*^13,53^*, NF-KBIA*^39,41,52^. Of those, two genes present in three copies in trisomic cells are Interferon Alpha and Beta Receptor Subunit 1 and 2 (*IFNAR1* and *IFNAR2*) that form a heterodimeric interferon receptor. IFNAR1 and IFNAR2 initiate the innate antiviral immune response, that leads to the phosphorylation of STAT1-STAT2, that dimerize and activate transcription of inflammatory genes in the nucleus. OAS1 and OAS2 are two HSA21 interactor proteins, activated by the interferon signaling pathway, leading to activation of the RNAseL that, in turn, leads to the degradation of the viral genome^54^. On the other hand, Nuclear Factor kappa B (NF-kB) inhibitor alpha, is activated by the inflammatory NF-kB signaling and inhibits the translocation of the same NF- kB into the nucleus. With the exception of IFNA1 expression, type I interferons (IFNA2 and IFNB1) were all upregulated, indicating that the axis IFNAR-STAT-OAS is upregulated in Down syndrome.

A recent paper shows that SARS-CoV-2 receptor ACE2 is an interferon-stimulated gene in human airway epithelial cells and is detected in specific cell subsets across tissues^55^. Therefore, we predict that virus entry might be significantly increased in DS patients that have both an increased interferon signaling and triplication of the protein S-priming through TMPRSS2.

Tightly connected to this pathway, the MAPK signaling acts as an integration point of several biological processes^56^. In this pathway we found 7 genes downregulated (*BCL2*^31,33,39,57^, *FOS*^32,42,49,57–59^, *IFITM2*^39,57,59^, *MAPK3*^39,42,59^, *MAPK10*^32,57^, *MAPK13*^33,49,60^, *MAPK14*^32,33^) and 5 upregulated (*MAPK1*^45,61^, *MAPK11*^13,62^, *IFITM1*^32,41,45,52^, *IFI27*^32,39,51,52,63^ and *BST2*^13,32,41,49,51,57^). Interestingly, some of these proteins, such as Interferon Induced Transmembrane protein 1 (IFITM1), Interferon Induced protein 27 (IFI27) and Bone marrow stromal antigen 2 precursor (BST2), are all interferon-induced proteins with antiviral properties. Specifically, IFITM1 is active against multiple viruses, including SARS-CoV^64^, preventing the viral fusion after endocytosis and the release of viral contents into the cytosol.

The viral Orf3a protein from SARS-CoV can bind TRAF3 and activate the NLRP3 inflammasome^65^, leading to the cytokine storm. Given the higher basal inflammation in DS we would have expected the inflammasome to be upregulated. Instead, we detected a strong downregulation of the *NLRP3* gene^39^, critical for maintenance of homeostasis against pathogenic infections^66^, along with lower levels of the gene for the NF- kB subunit *RELA*. Actually, even if the IFN-I signaling in the beginning induces an antiviral response, it eventually exerts an anti-inflammatory action inhibiting the NLRP3 inflammasome through STAT1. Although this could potentially be beneficial in later stage to shut inflammation down, it could also be one of the reasons why DS patients with influenza often manifest bacterial infection complications^67^.

Moreover, DS individuals could be more susceptible to late onset complication such as lung fibrosis upon COVID19 infection, because they upregulate some of the cytokines responsible for the so-called “cytokine storm”. Specifically, we detected upregulation of the chemokine CXCL10^45^ that induces chemotaxis and stimulation of monocytes, and of Interleukin 10^32^. IL10 is an anti-inflammatory cytokine necessary for regulated resolution of inflammation. However, IL10 recruits fibrocytes and activates M2 macrophages in a CCL2/CCR2 axis and mice overexpressing IL10 show lung fibrosis^68^.

Finally, we found upregulated ZAP70^32^ a tyrosine kinase that regulates motility, adhesion and cytokine expression of mature T-cells, as well as thymocyte development. A recent study found the SARS-CoV-2 nsp9 and nsp10 interact with NKRF^69^, that inhibits IL-8 and IL-6 induction by competing with NF-КB for promoter binding. This interaction would lead to IL8/IL6 induction, and, among other, inhibition of ZAP70.

### Apoptosis is inhibited in DS

As regards apoptosis, it is known that the apoptotic effect of SARS-CoV is mediated by its M protein through inhibition of the proto-oncogene AKT1^70^. Interestingly, AKT1 is a HSA21 interactor and is consistently upregulated^61^. We therefore speculate that trisomic cells might be less sensitive to the apoptotic effect of coronaviruses.

Apoptosis can also be induced through endoplasmic reticulum (ER) stress. As a matter of fact, coronavirus replication is associated with the endoplasmic reticulum and this often induces ER stress, with subsequent activation of the unfolded protein response (UPR). UPR leads to the blockade of protein synthesis, and subsequent apoptosis through EIF2AK2^71^. EIF2AK2 is an interferon-induced serine-threonine protein kinase (PKR), that, once activated by the viral RNA, phosphorylates and inhibits the initiation factor eIF2α, preventing viral replication. The coronavirus’ non-structural related protein 15 is an endoribonuclease that can inhibit EIF2AK2 preventing a premature block of protein synthesis upon viral infection^72^. However, in DS, *EIF2AK2* levels are elevated^32,40–42^, and therefore, once again, trisomic cells might be more resistant to this inhibition. Supporting this, the serine/threonine-protein phosphatase PP1-alpha catalytic subunit PPP1CA, one of the three catalytic subunits of protein phosphatase 1 (PP1), is also downregulated in DS^32,42^, leading again to the translation shutoff mediated by phosphorylated eIF2α.

### Host proteins interacting with viral proteins are enriched in viral life cycle and tight junction

When we analyzed at the portion of DS-SARS-CoV-2 network of host proteins interacting with viral proteins (squared nodes) we found that they were enriched in two main categories: the biological process “viral life cycle” and the KEGG pathway “tight junction”^30^. Among the first are worth mentioning the nucleoporin *NUP62*^33,39^ and *NUP210*^39,42^, that are downregulated in DS. Upon viral infection, nucleoporins are normally degraded to suppress innate immune responses, and improve viral replication and transmission^73^. This may indicate altered virus-host interactions in DS leading to more efficient viral innate immune evasion^74^. As regards the tight junction pathway, we detected 4 genes upregulated and 2 downregulated in DS. Even though the net effect of these deregulations cannot be predicted, interestingly, many viruses, including coronaviruses, disrupt epithelial tight junctions of the respiratory tract that serve as a barrier to invaders^75^.

Another interesting protein is a disintegrin and metalloproteinase with thrombospondin motifs 1 (ADAMTS1) that interacts with the viral helicase/triphosphatase nsp13. While the significance of this interaction is unknown, it is known that *ADAMTS1* is triplicated and upregulated in DS, both in blood datasets and in DS lung^13,29,39,42,76^, where it contributes to the global anti-angiogenic milieu leading to higher risk for developing pulmonary arterial hypertension (PAH) in infants with DS. Moreover, it induces fibrosis in myocardial viral infection^77,78^.

### Antiviral properties of EGCG

Compared to SARS-CoV, SARS-CoV2 seems to be much more infectious. This might be due to the ability of the virus to be primed not only by TMPRSS2 but also by FURIN.

EGCG, the main polyphenol of green tea, showed some beneficial effects on cognition in a phase II clinical trial with DS individuals^79^. Interestingly, EGCG perfectly fits in the FURIN pocket and is therefore predicted to be a FURIN inhibitor^80–82^. Concordantly, lipophilic EGCG derivatives showed antioxidant and antiviral properties^83^. Therefore, the use of EGCG in individuals with DS might be beneficial in the context of this pandemic. However, we doubt that this could be enough as a treatment or prevention, given TMPRSS2 triplication.

## Discussion

COVID-19 is a new disease and there is limited information regarding risk factors for infection and worse prognosis. It has been suggested that individuals with DS should be considered a vulnerable at risk population for severe COVID-19, as recognized by the Trisomy 21 Research Society recommendations (www.t21rs.org). Based on currently available information, DS individuals are in fact easily predicted to be at higher risk for severe illness from COVID-19, as they present increased prevalence of medical comorbidities associated with worse prognosis, including diabetes, cardiovascular disease, and respiratory problems. Besides, individuals with DS present clinical history of infections, increased rates of hospitalization upon respiratory viral infections, and higher mortality rates from pneumonia and sepsis and the immune dysregulation caused by trisomy 21 may result in an exacerbated cytokine release syndrome relative to the euploid population. Indeed, previous work reported increased pro-inflammatory markers in plasma of a DS mouse model (Ts65Dn)^84^ and intrinsic lymphopenia^85^ and elevated levels of pro-inflammatory cytokines, IL-6, MCP-1, IL-22 and TNF-α in individuals with DS^13^.

**Figure 4:**
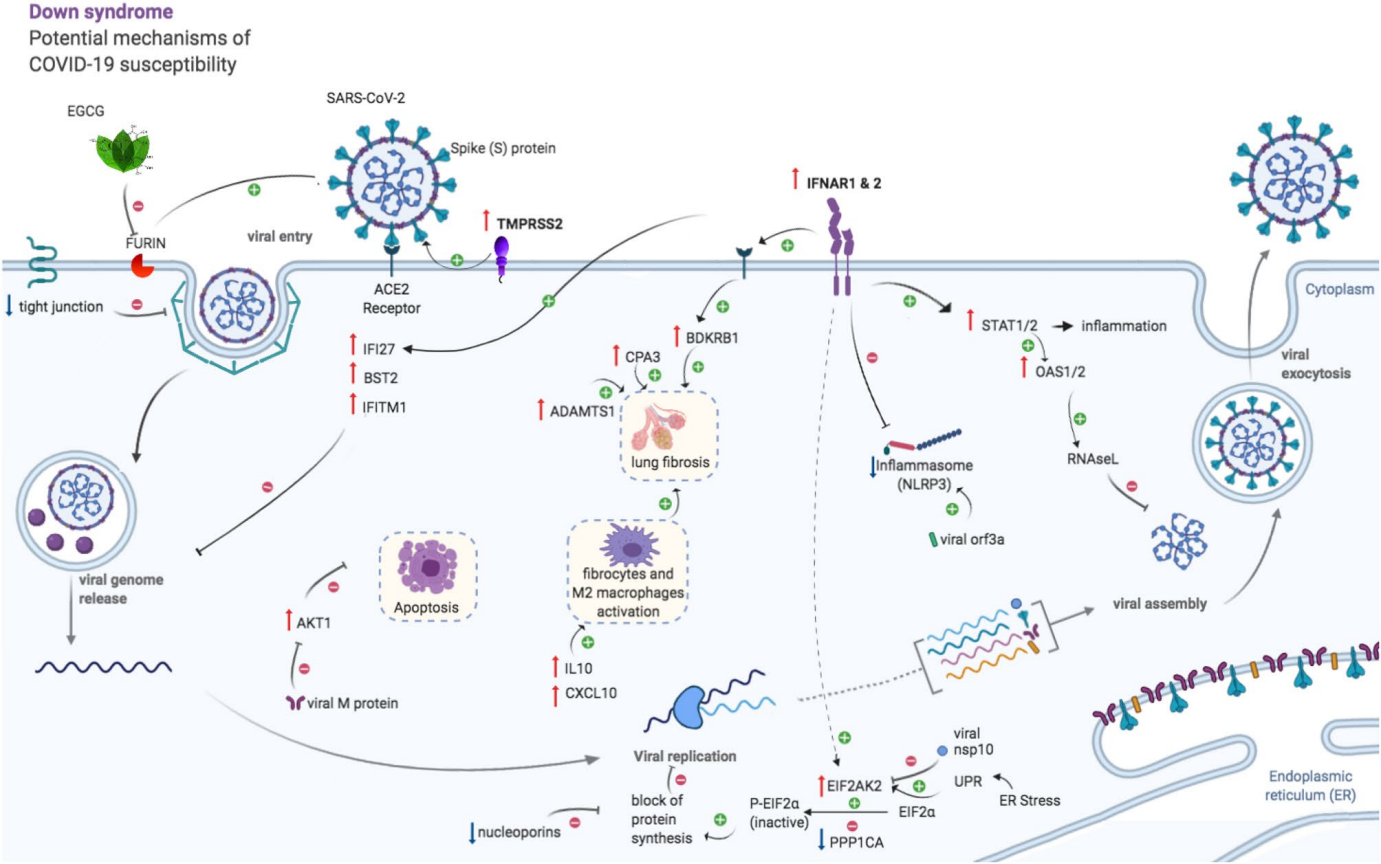
Summary of the molecular processes during COVID-19 infections that are affected in DS. The figure recapitulates the pathways affected in DS, and how they contribute to Sars-Cov-2 infection and the severity of COVID-19. Mechanisms contributing to viral entry: viral entry could be facilitated in DS thanks to *TMPRSS2* triplication that leads to increased activation of the viral S-protein and also thanks to tight junctions’ downregulation, since tight junctions hamper virus’ endocytosis. Another protease able to prime the S-protein is FURIN, which can be inhibited by EGCG, a polyphenol that has been used in DS individuals. Mechanisms involved in inflammation and viral replication: triplication of *IFNAR1/2 *results in activation of the interferon (IFN) I response and subsequently of the JAK/STAT/OAS pathway, that in turn, leads both to increased inflammation but also to activation of the RNAseL that could degrade the viral RNA. We detected upregulation in DS of the interferon-induced antiviral proteins *IFI27, BST2, IFITM1*, that inhibit viral genome release. ER stress, the unfolded protein response, and the IFN signaling lead to upregulation of *EIF2AK2* that phosphorylate/inactivated EIF2α leading to the block of protein synthesis. The virus inhibits EIF2AK2 to allow viral replication, while in DS upregulation of *EIF2AK2* together with downregulation of the phosphatase *PPP1CA* might block protein synthesis. However, the downregulation of nucleoporins in DS might instead favor viral replication. The viral protein M inhibits AST1 leading to apoptosis which could be counteracted in DS by *AKT1* upregulation. Mechanisms involved in the severity of COVID-19: the IFN signaling also leads to increased bradykinin signaling (via *ACE2* upregulation), that together with higher levels of CPA3 and ADAMTS1 can lead to lung fibrosis. The IFN signaling eventually downregulates the NLRP3 inflammasome (that is normally activated by the viral orf3a) which is downregulated in DS, leading to higher susceptibility to secondary bacterial infections. Finally, the cytokine storm could be potentiated in DS as a result of increased levels of the chemokine *CXCL10* and *IL10* that leads to increased fibrocytes and M2 macrophages activation leading to lung fibrosis. Upper red arrows indicate that the given gene is upregulated, blue down arrow, downregulated.

Our analysis showed that a number of host-virus interactions are altered in DS, including viral entry and viral spreading, that could be significantly different in DS individuals compared to the general population (Fig. 4, Table 1). Viral entry could be facilitated in DS thanks to TMPRSS2 triplication that leads to increased activation of the viral S protein and also thanks to tight junctions’ downregulation, since tight junctions hamper virus’ endocytosis.

**Table 1:** COVID19 related pathways affected in DS.

Pathways	Source	HSA21 genes	HSA21 interactors	Consistently DE	Upregulated genes	Downregulated genes	Affected process
Type I interferon	WikiPathway	IFNAR1, IFNAR2	CHUK, IKBKB, NFKBIA, MAPK8, MAPK14, JUN, NFKB1, IFNA1, IFNAR1, JAK1, IFNAR2, TYK2, STAT1, STAT2, OAS1, OAS2, IRF3, FOS, IRF9, OAS3	IFNAR1, IFNAR2, EIF2AK2, FOS	NFKBIA, IFNAR1, IFNAR2, STAT1, STAT2, EIF2AK2, OAS1, OAS2	MAPK14, IFNA1, FOS	Inflammation, Viral genome release
MAPK	WikiPathway		MAPK3, MAPK1, MAPK11, MAPK12, MAPK13, MAPK14, MAPK8, MAPK9, MAPK10, JUN, FOS, IFITM1, IFITM2, BCL2, IFITM3	FOS, IFI27, BST2, IFITM2, BCL2	MAPK1, MAPK11, IFI27, BST2, IFITM1	MAPK3, MAPK13, MAPK14, MAPK10, FOS, IFITM2, BCL2	Viral fusion after endocytosis, release of viral content
ACE2	WikiPathway	TMPRSS2	BDKRB1, AGT, AGTR1, TGFB1, CTSB	CTSG, CPA3	BDKRB1, AGT, CTSG, CPA3, TMPRSS2	TGFB1, CTSB	Viral entry, bradykinin signaling
Apoptosis	WikiPathway		BCL2, CASP8, CASP3, MAPK11, MAPK12, MAPK13, MAPK14, AKT1	BCL2	MAPK11, AKT1	BCL2, MAPK13, MAPK14	Viral mediated apoptosis
ER stress	WikiPathway		MAPK8, MAPK9, MAPK10	PPP1CA, EIF2AK2	EIF2AK2	PPP1CA, MAPK10	Protein synthesis, viral replication
Inflammasome	WikiPathway		NFKB1, NLRP3, RELA			NLRP3, RELA	Secondary bacterial infections
Lung fibrosis	WikiPathway		CXCL8, IL10, CXCL10		IL10, CXCL10		Cytokine storm
nsp9-nsp10 mediated pathogenesis	WikiPathway		CXCL8, ZAP70		ZAP70		Inflammation
Ubiquitination	WikiPathway		NAE1, CBFB		CBFB		Viral replication
Autophagy	WikiPathway			ULK2	ULK2		Viral replication
Viral life cycle	Gene, Ontology	TMPRSS2	SCARB1, NUP210, IDE, NUP54, ITGB1, NUP62, NUP214, RAE1, NUP88, OAS3, CXCL8, BCL2, IFITM1, CTSB, IFITM3, IFITM2, OAS1	LARP1, STOM, EIF2AK2, BST2, IFI27, BCL2, IFITM2	SCARB1, ITGB1, LARP1, STOM, NUP214, EIF2AK2, BST2, IFI27, IFITM1, OAS1, TMPRSS2	NUP210, NUP62, BCL2, CTSB, IFITM2	Viral replication
Tight junction	KEGG		ITGB1, RAB8A, PRKACA, RHOA, MAPK10, MAPK8, JUN, MAPK9	RDX	ITGB1, PRKACA, RDX, RHOA	RAB8A, MAPK10	Viral endocytosis

Another protease able to prime the S protein is FURIN, which can be inhibited by EGCG, a polyphenol that has been used in DS individuals. Other transcriptional disturbances detected in DS could influence inflammation and viral replication such as triplication of *IFNAR1/2* results in activation of the interferon (IFN) I response and subsequently of the JAK/STAT/OAS pathway, that in turn, leads both to increased inflammation but also to activation of the RNAseL that could degrade the viral RNA. We detected upregulation in DS of the interferon-induced antiviral proteins *IFI27, BST2, IFITM1*, that inhibit viral genome release. ER stress, the unfolded protein response, and the IFN signaling lead to upregulation of *EIF2AK2* that phosphorylate/inactivated EIF2α leading to the block of protein synthesis. The virus inhibits EIF2AK2 to allow viral replication, while in DS upregulation of *EIF2AK2* together with downregulation of the phosphatase *PPP1CA* might block protein synthesis. However, the downregulation of nucleoporins in DS might, instead, favor viral replication. The viral protein M inhibits AST1 leading to apoptosis which could be counteracted in DS by *AKT1* upregulation.

Interestingly, some molecular processes seem to be protective against Covid-19 in DS. Interferon signaling, for example, leads to downregulation of the NLRP3 inflammasome. However, this might make DS patients more susceptible to post-viral complications, such as bacterial infections. Other potentially protective molecular signatures we detected in DS are:

1. the upregulation of antiviral proteins such as IFITM1, IFI27 and BST2, probably mediated by interferon I signaling
2. resistance to the apoptotic effect of coronaviruses (that might favor however they replication in a first moment)
3. Resistance to the prevention of the block of protein synthesis mediated by the virus

At first glance the triplication of *IFNAR1* and *IFNAR2,* with consequent upregulation of the anti-viral Type I interferon signaling, might suggest higher defenses in DS individuals in a first stage of the infection. However, this might be overcome by overexpression of *TMPRSS2*, with subsequent priming of the viral S-protein for the binding with the ACE2 receptors. *TMPRSS2* overexpression was detected in several tissues, which may also favor the appearance of a number of extra-pulmonary COVID-19 effects. Besides, the interferon signaling itself leads to upregulation of the *ACE2* receptors in airway epithelial cells as shown in the general population.

Both these processes would lead to a facilitated virus entry. Unfortunately, in our datasets we did not have the transcriptome of airway epithelial cells to check if in DS there are more ACE2 receptors. Moreover, the upregulation of the bradykinin receptor B1 and of the metalloprotease CPA3 in DS, might lead to an increased susceptibility to ARDS.

Trisomy of HSA21 could also facilitate some mechanisms involved in the severity of COVID-19. Once the coronavirus spreads enough, the cytokine storm in DS might have more severe consequence due to higher levels of chemokines (such as CXCL10), inducing chemotaxis and stimulation of monocytes, and Interleukin 10 (IL10), that even though it is classified as an anti-inflammatory interleukin, it recruits fibrocytes and activates M2 macrophages and may lead to later lung fibrosis.

Moreover, the upregulation of the IFN signaling leads to increased bradykinin signaling (via ACE2 upregulation), that together with higher levels of *CPA3 *and *ADAMTS1 *can lead to lung fibrosis. The IFN signaling eventually downregulates the NLRP3 inflammasome (that is normally activated by the viral orf3a) which is downregulated in DS, leading to higher susceptibility to secondary bacterial infections.

We based our analysis on the pathways that have been included in the special session of WikiPathways dedicated to COVID-19 pathways. However, other processes might be also relevant in DS individuals in the context of COVID-19. Moreover, gene deregulation can be quite variable across tissues, even though HSA21 is triplicated in every cells (with the exception of mosaics). For this reason, when we describe a gene as “upregulated” or “downregulated” we refer at the mean fold change across all the examined comparisons. This is influenced by the datasets that we used for the analysis (where blood and brain tissue are overrepresented).

Another limitation is that, since our data origin from different tissue/cell types, and both from mouse and human it was not possible to assess the age-dependent risk. This would have been interesting, especially in lights of the results of the Trisomy 21 Research Society’s survey showing how risk for fatal outcome is increased from age 40 in DS patients affected with COVID-19 (https://www.t21rs.org/results-from-covid-19-and-down-syndrome-survey/). Future studies on DS patient with COVID-19 will get deeper insight on the severity of this pandemic in its various phases in the context of trisomy 21.

## Conclusion

We detected both COVID-19 protective and risk factors among HSA21 genes and interactors and/or DS deregulated genes that might affect the susceptibility of individuals with DS. At the infection stage, individuals with DS might be more susceptible to infection due to triplication of *TMPRSS2*. However, the upregulation of the anti-viral interferon I signaling in DS might increase anti-viral response, inhibiting viral genome release, viral replication and viral assembly. In the pro-inflammatory immunopathogenic phase of the infection, upregulation of inflammatory genes might favor the typical cytokine storm of COVID-19. Finally, the strong downregulation of *NLRP3*, critical for maintenance of homeostasis against pathogenic infections, may favour bacterial infection complications. On balance, we consider that DS individuals might be particularly at risk during this pandemic, both at the stage of infection and for the prognosis once the cytokine storms begin (Fig. 4, Table 1). However, this increased risk may apply predominantly to DS individuals older than 40 years of age or those with significant comorbidities. Future epidemiological data would help to investigate this hypothesis.

## Supplementary Information

Supplementary Information.

## Data Availability

Available upon request.

## Abbreviations

ACE2: Angiotensin I converting enzyme 2
ADAMTS1: A disintegrin and metalloproteinase with thrombospondin motifs 1
ARDS: Acute respiratory distress syndrome
BST2: Bone marrow stromal antigen 2 precursor
BDKRB1: Bradykinin receptor B1
DS: Down syndrome
ER: Endoplasmic reticulum
HSA21: Human chromosome 21
iPSC: Induced pluripotent stem cells
IFNAR1 and IFNAR2: Interferon Alpha and Beta Receptor Subunit 1 and 2
IFI27: Interferon induced protein 27
IFITM1: Interferon induced transmembrane protein 1
IL10: Interleukin 10
NFKBIA: Nuclear factor kappa B inhibitor alpha
PAH: Pulmonary arterial hypertension
SARS-CoV-2: Sever acute respiratory syndrome coronavirus 2
S: Spike
UPR: Unfolded protein response
