# Supplementary material for: Network analysis of Down syndrome and SARS-CoV-2 identifies risk and protective factors for COVID-19
Source: Sci Rep. 2021 Jan 21;11:1930. doi: 10.1038/s41598-021-81451-w (PMC7820501; doi:10.1038/s41598-021-81451-w)
Supplement: Supplementary file 1 — Supplementary Information. [file 41598_2021_81451_MOESM1_ESM.pdf]

Supplementary Material

Network analysis of Down syndrome and SARS-CoV-2 identifies risk and protective factors for COVID-19

Ilario De Toma[1] and Mara Dierssen[1,2,3]

[1] Centre for Genomic Regulation (CRG), The Barcelona Institute of Science and Technology, Barcelona, Spain

[2] Universitat Pompeu Fabra (UPF), Barcelona, Spain

[3] Biomedical Research Networking Center on Rare Diseases (CIBERER), Institute of Health Carlos III, Madrid, Spain.

Supplementary Figure 1. DS-SARS-CoV-2 network colored with mean log2FC. Same network from figure 1 but with the node color according to the mean log2FC in studies comparing Down syndrome versus control group. Red indicates a positive log2FC, blue a negative log2FC, when this information is not available the node is in gray.

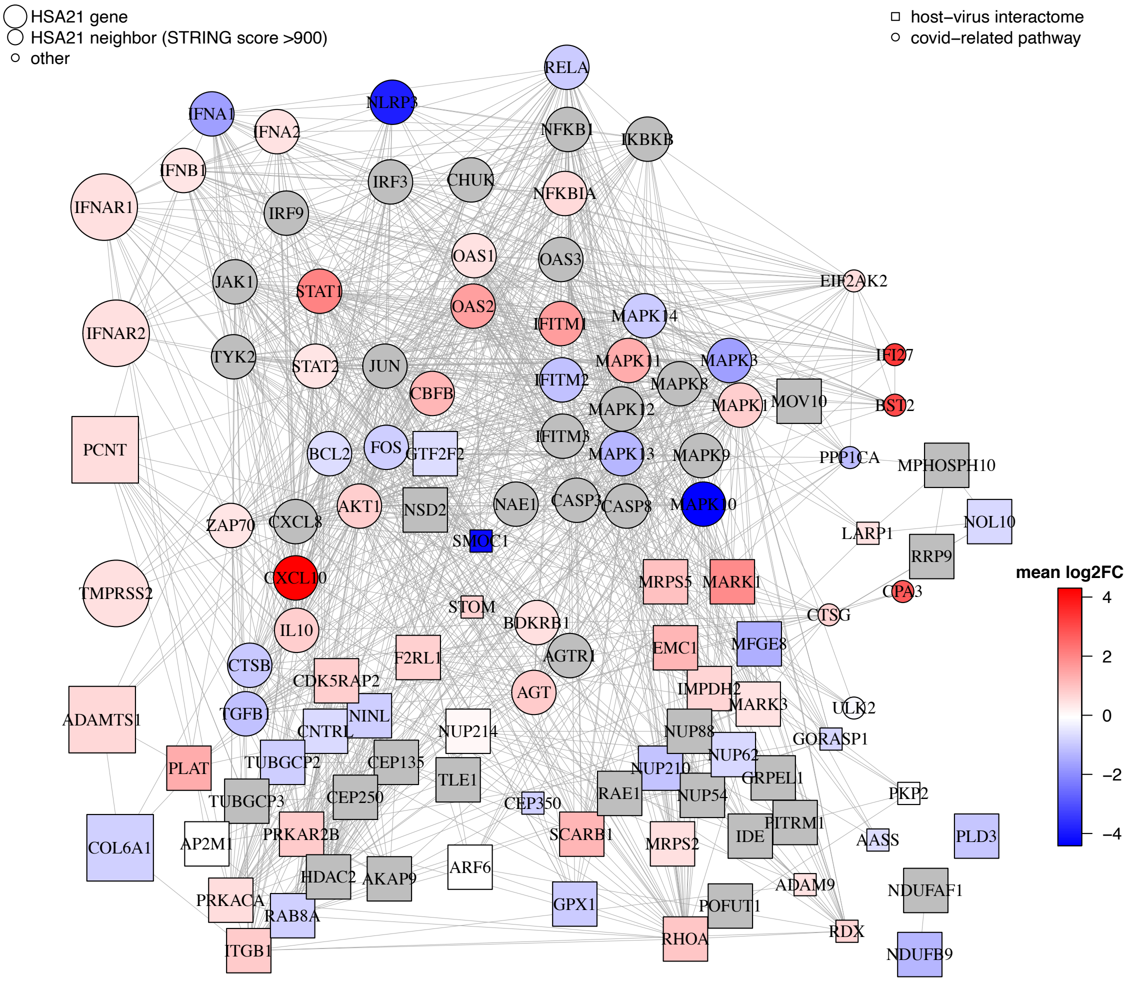

Supplementary Table 1. List of datasets that were used in this analysis, with their references.

Supplementary_table_1

Name	GEO/ArrayExpress	SRA_study	Species	Model	Platform	Cell_type	Tissue	Sample_type	PMID	DE_p.1	DE_p.05	DE_p.01	DE_p.001	Design	Df
GSE52249	GSE52249	SRP032928	Homo sapiens	NA	Illumina HiSeq 2000	iPSCs	fibroblasts	iPSCs	24375627	11475	9826	7218	5081	4 euploid; 3 trisomy 21	5
GSE55504	GSE55504	SRP039348	Homo sapiens	NA	Illumina HiSeq 2000	Primary Fibroblasts	umbilical cord tissue	Fibroblasts	24740065	198	125	55	32	4 euploid; 4 trisomy 21	6
GSE55504_fibroblasts	GSE55504	SRP039348	Homo sapiens	NA	Illumina HiSeq 2000	Primary Fibroblasts	Primary skin fibroblasts	Fibroblasts	24740065	436	282	119	66	8 euploid; 8 trisomy 21	14
GSE55504_ipsc	GSE55504	SRP039348	Homo sapiens	NA	Illumina HiSeq 2000	iPSCs	Primary fetal skin fibroblasts	iPSCs	24740065	0	0	0	0	2 euploid; 1 trisomy 21	1
GSE55504_mef	GSE55504	SRP039348	Mus musculus	Ts65Dn	Illumina HiSeq 2000	MEFs	14.5-day-old mouse embryos	Fibroblasts	24740065	0	0	0	0	1 euploid; 1 trisomy 21	0
GSE42142	GSE42142	SRP017123	Homo sapiens	NA	Illumina Genome Analyzer IIx	CVS	Fetal placenta tissue	Placenta/amnios	23754950	149	87	31	9	4 euploid; 4 trisomy 21	6
GSE64840	GSE64840	SRP051977	Mus musculus	Ts65Dn	Illumina HiSeq 2500	MEFs	18.5-day-old embryos	Fibroblasts	26664707	3053	2521	1847	1319	3 euploid; 3 trisomy 21	4
GSE101942_ipsc	GSE101942	SRP113668	Homo sapiens	NA	Illumina HiSeq 2000	iPSCs	iPSCs	iPSCs	29584757	1590	1267	861	556	3 euploid; 3 trisomy 21	4
GSE101942_neurons	GSE101942	SRP113668	Homo sapiens	NA	Illumina HiSeq 2000	iPSC-derived Neurons	differentiation length: 40 days	Neurons	29584757	0	0	0	0	3 euploid; 3 trisomy 21	4
GSE6283_amnio	GSE6283	NA	Homo sapiens	NA	hgu133plus2	amniocytes	Fetuses	Placenta/amnios	18253026	18	1	0	0	3 euploid; 3 trisomy 21	4
GSE6283_cvs	GSE6283	NA	Homo sapiens	NA	hgu133plus2	CVS	Fetuses	Placenta/amnios	18253026	893	338	61	9	6 euploid; 3 trisomy 21	7
GSE1294	GSE1294	NA	Mus musculus	Ts1Cje	mgu74av2	Whole Brain	Whole Brain	whole brain	15138197	20	15	10	8	6 euploid; 6 trisomy 21	10
GSE1294b	GSE1294	NA	Mus musculus	Ts1Cje	mgu74bv2	Whole Brain	Whole Brain	whole brain	15138197	17	14	8	6	6 euploid; 6 trisomy 21	10
GSE1611	GSE1611	NA	Mus musculus	Ts1Cje	mgu74av2	Cerebellum	Cerebellum	Cerebellum	15590701	27	18	10	4	6 euploid; 6 trisomy 21	10
GSE5390	GSE5390	NA	Homo sapiens	NA	hgu133a	Dorsolateral prefrontal cortex	post-mortem Dorsolateral prefrontal cortex	Cortex	17950572	7119	5359	2133	79	8 euploid; 7 trisomy 21	13
EMEXP409	E-MEXP-409	NA	Mus musculus	Tc1	hgu133a	whole embryo	whole embryo	whole embryo	16179473	1394	1293	516	0	1 euploid; 2 trisomy 21	1
GSE1397.crb	GSE1397	NA	Homo sapiens	NA	hgu133a	Cerebrum	Cerebrum	Cerebrum	16420667	0	0	0	0	4 euploid; 4 trisomy 21	6
GSE1397.crbl	GSE1397	NA	Homo sapiens	NA	hgu133a	Cerebellum	Cerebellum	Cerebellum	16420667	8	0	0	0	3 euploid; 3 trisomy 21	4
GSE1397.astro	GSE1397	NA	Homo sapiens	NA	hgu133a	astrocytes	Cerebrum	astrocytes	16420667	9	6	4	1	2 euploid; 2 trisomy 21	2
GSE1397.heart	GSE1397	NA	Homo sapiens	NA	hgu133a	Heart	Heart	Heart	16420667	41	24	4	0	2 euploid; 2 trisomy 21	2
GSE9762	GSE9762	NA	Homo sapiens	NA	hgu133plus2	Primary Fibroblasts	Primary skin fibroblasts	Fibroblasts	NA	363	57	0	0	5 euploid; 5 trisomy 21	8
GSE16176	GSE16176	NA	Homo sapiens	NA	hgu133plus2	amniotic fluid	amniotic fluid	Placenta/amnios	19474297	0	0	0	0	7 euploid; 7 trisomy 21	12
EMEXP654	E-MEXP-654	NA	Mus musculus	transchromosomic cells 47-1	mgu74av2	mESCs	mESCs	ESCs	20569505	2673	1678	503	104	4 euploid; 4 trisomy 21	6
GSE35561	GSE35561	NA	Homo sapiens	NA	HuGene-1_0-st	iPSCs	embryos	iPSCs	23045704	0	0	0	0	3 euploid; 3 trisomy 21	4
GSE39159	GSE39159	NA	Mus musculus	Ts65Dn	Affymetrix Mouse Gene 1.0 ST Array	Soleus muscles	Soleus muscles	Muscle	23115123	1828	6	1	1	4 euploid; 4 trisomy 21	6
GSE110064	GSE110064	NA	Homo sapiens	NA	hgu133plus2	B-cells	iPSCs	B-cells	29789608	0	0	0	0	3 euploid; 3 trisomy 21	4
GSE99135dp16.E15	GSE99135	NA	Mus musculus	Dp16	Affymetrix Mouse Gene 1.0 ST Array	dp16.E15	embryonic forebrain (E15)	Forebrain	29716957	3566	2532	1155	396	6 euploid; 6 trisomy 21	8
GSE99135Ts65Dn.E15	GSE99135	NA	Mus musculus	Ts65Dn	Affymetrix Mouse Gene 1.0 ST Array	Ts65Dn.E15	embryonic forebrain (E15)	Forebrain	29716957	180	144	102	76	7 euploid; 6 trisomy 21	11
GSE99135Ts1Cje.E15	GSE99135	NA	Mus musculus	Ts1Cje	Affymetrix Mouse Gene 1.0 ST Array	Ts1Cje.E15	embryonic forebrain (E15)	Forebrain	29716957	148	108	72	53	5 euploid; 5 trisomy 21	8
GSE99135dp16.Crblm	GSE99135	NA	Mus musculus	Dp16	Affymetrix Mouse Gene 1.0 ST Array	dp16.crblm	Cerebellum	Cerebellum	29716957	77	70	52	27	5 euploid; 5 trisomy 21	8
GSE99135Ts65Dn.Crblm	GSE99135	NA	Mus musculus	Ts65Dn	Affymetrix Mouse Gene 1.0 ST Array	Ts65Dn.crblm	Cerebellum	Cerebellum	29716957	161	137	98	76	5 euploid; 5 trisomy 21	8
GSE99135Ts1Cje.Crblm	GSE99135	NA	Mus musculus	Ts1Cje	Affymetrix Mouse Gene 1.0 ST Array	Ts1Cje.crblm	Cerebellum	Cerebellum	29716957	299	195	114	86	5 euploid; 5 trisomy 21	8
GSE99135dp16.Crtx	GSE99135	NA	Mus musculus	Dp16	Affymetrix Mouse Gene 1.0 ST Array	dp16.crtx	Cortex	Cortex	29716957	64	47	15	2	5 euploid; 5 trisomy 21	5
GSE99135Ts65Dn.Crtx	GSE99135	NA	Mus musculus	Ts65Dn	Affymetrix Mouse Gene 1.0 ST Array	Ts65Dn.crtx	Cortex	Cortex	29716957	148	115	86	72	5 euploid; 5 trisomy 21	8
GSE99135Ts1Cje.Crtx	GSE99135	NA	Mus musculus	Ts1Cje	Affymetrix Mouse Gene 1.0 ST Array	Ts1Cje.crtx	Cortex	Cortex	29716957	79	74	64	24	5 euploid; 6 trisomy 21	9
GSE99135dp16.Hpcmp	GSE99135	NA	Mus musculus	Dp16	Affymetrix Mouse Gene 1.0 ST Array	dp16.Hpcmp	Hippocampus	Hippocampus	29716957	233	129	63	37	5 euploid; 5 trisomy 21	8
GSE99135Ts65Dn.Hpcmp	GSE99135	NA	Mus musculus	Ts65Dn	Affymetrix Mouse Gene 1.0 ST Array	Ts65Dn.Hpcmp	Hippocampus	Hippocampus	29716957	112	95	75	60	5 euploid; 5 trisomy 21	8
GSE99135Ts1Cje.Hpcmp	GSE99135	NA	Mus musculus	Ts1Cje	Affymetrix Mouse Gene 1.0 ST Array	Ts1Cje.Hpcmp	Hippocampus	Hippocampus	29716957	250	153	76	56	7 euploid; 5 trisomy 21	10
GSE69210	GSE69210	NA	Homo sapiens	NA	Agilent-026652 Whole Human Genome Microarray 4x44K v2	Thymus	Thymus	Thymus	26848775	250	141	42	2	10 euploid; 10 trisomy 21	18
GSE84887	GSE84887	NA	Homo sapiens	NA	hgu133plus2	NPCs	Neural Progenitor cells	NPCs	27618722	1	0	0	0	3 euploid; 5 trisomy 21	6
GSE83449	GSE83449	NA	Homo sapiens	NA	hgu133a	AMKL	Blood	Blood/bone marrow	16492768	5341	5157	2625	1388	31 euploid; 22 trisomy 21	50
GSE59630dfc.fetal	GSE59630	NA	Homo sapiens	NA	HuEx-1_0-st	DFC	fetal dorsolateral prefrontal cortex (DFC)	Cortex	26924435	135	91	50	33	2 euploid; 2 trisomy 21	2
GSE59630dfc.postnatal	GSE59630	NA	Homo sapiens	NA	HuEx-1_0-st	DFC	postnatal dorsolateral prefrontal cortex (DFC)	Cortex	26924435	121	48	14	8	7 euploid; 7 trisomy 21	12
GSE59630dfc.adult	GSE59630	NA	Homo sapiens	NA	HuEx-1_0-st	DFC	adult dorsolateral prefrontal cortex (DFC)	Cortex	26924435	2608	1624	673	209	3 euploid; 3 trisomy 21	4
GSE59630cbc.postnatal	GSE59630	NA	Homo sapiens	NA	HuEx-1_0-st	CBC	postnatal cerebellar cortex (CBC)	Cerebellum	26924435	1582	989	377	139	8 euploid; 8 trisomy 21	14
GSE59630cbc.adult	GSE59630	NA	Homo sapiens	NA	HuEx-1_0-st	CBC	adult cerebellar cortex (CBC)	Cerebellum	26924435	817	309	19	2	2 euploid; 2 trisomy 21	2
GSE59630hip.postnatal	GSE59630	NA	Homo sapiens	NA	HuEx-1_0-st	HIP	postnatal hippocampus (HIP)	Hippocampus	26924435	0	0	0	0	2 euploid; 2 trisomy 21	2
GSE59630hip.adult	GSE59630	NA	Homo sapiens	NA	HuEx-1_0-st	HIP	adult hippocampus (HIP)	Hippocampus	26924435	9	6	6	2	1 euploid; 1 trisomy 21	0
GSE59630v1c.fetal	GSE59630	NA	Homo sapiens	NA	HuEx-1_0-st	V1C	fetal primary visual cortex (V1C)	Cortex	26924435	2517	695	62	9	2 euploid; 2 trisomy 21	2
GSE59630v1c.postnatal	GSE59630	NA	Homo sapiens	NA	HuEx-1_0-st	V1C	postnatal primary visual cortex (V1C)	Cortex	26924435	368	177	75	15	7 euploid; 7 trisomy 21	12
GSE59630v1c.adult	GSE59630	NA	Homo sapiens	NA	HuEx-1_0-st	V1C	adult primary visual cortex (V1C)	Cortex	26924435	686	301	22	0	2 euploid; 2 trisomy 21	2
GSE59630itc.postnatal	GSE59630	NA	Homo sapiens	NA	HuEx-1_0-st	ITC	postnatal inferior temporal cortex (ITC)	Cortex	26924435	2621	720	2	1	3 euploid; 3 trisomy 21	4
GSE59630itc.adult	GSE59630	NA	Homo sapiens	NA	HuEx-1_0-st	ITC	adult inferior temporal cortex (ITC)	Cortex	26924435	2308	1284	264	31	2 euploid; 2 trisomy 21	2
GSE59630stc.postnatal	GSE59630	NA	Homo sapiens	NA	HuEx-1_0-st	STC	postnatal posterior superior temporal cortex (STC)	Cortex	26924435	0	0	0	0	1 euploid; 1 trisomy 21	0
GSE59630IPC.postnatal	GSE59630	NA	Homo sapiens	NA	HuEx-1_0-st	IPC	postnatal posterior inferior pariteal cortex (IPC)	Cortex	26924435	0	0	0	0	1 euploid; 1 trisomy 21	0
GSE59630s1c.postnatal	GSE59630	NA	Homo sapiens	NA	HuEx-1_0-st	S1C	postnatal primary somatosensory cortex (S1C)	Cortex	26924435	0	0	0	0	1 euploid; 1 trisomy 21	0
GSE59630mfc.postnatal	GSE59630	NA	Homo sapiens	NA	HuEx-1_0-st	MFC	postnatal medial prefrontal cortex (MFC)	Cortex	26924435	0	0	0	0	1 euploid; 1 trisomy 21	0
GSE59630vfc.postnatal	GSE59630	NA	Homo sapiens	NA	HuEx-1_0-st	VFC	postnatal ventral prefrontal cortex (VFC)	Cortex	26924435	2	0	0	0	5 euploid; 5 trisomy 21	8
GSE59630vfc.adult	GSE59630	NA	Homo sapiens	NA	HuEx-1_0-st	VFC	adult ventral prefrontal cortex (VFC)	Cortex	26924435	0	0	0	0	2 euploid; 2 trisomy 21	2
GSE59630ofc.postnatal	GSE59630	NA	Homo sapiens	NA	HuEx-1_0-st	OFC	postnatal orbital prefrontal cortex (OFC)	Cortex	26924435	6009	4234	852	6	4 euploid; 4 trisomy 21	6
GSE59630ofc.adult	GSE59630	NA	Homo sapiens	NA	HuEx-1_0-st	OFC	adult orbital prefrontal cortex (OFC)	Cortex	26924435	186	45	5	1	2 euploid; 2 trisomy 21	2
GSE58463Ts2Yah	GSE58463	NA	Mus musculus	Ts2Yah	Illumina MouseWG-6 v2.0	gastrocnemius muscles	gastrocnemius muscles	Muscle	NA	3	2	0	0	5 euploid; 6 trisomy 21	9
GSE65055	GSE65055	NA	Homo sapiens	NA	HuGene-2_0-st	CVS	placenta	Placenta/amnios	NA	0	0	0	0	5 euploid; 5 trisomy 21	8
GSE70102	GSE70102	NA	Homo sapiens	NA	hgu133plus2	maternal fetal interface	placenta	Placenta/amnios	27328057	6242	3980	1067	34	4 euploid; 8 trisomy 21	10
GSE68074	GSE68074	NA	Mus musculus	Ts65Dn	Agilent-028005 SurePrint G3 Mouse GE 8x60K Microarray	Hippocampus	Hippocampus	Hippocampus	26546125	0	0	0	0	4 euploid; 3 trisomy 21	5
GSE36787	GSE36787	NA	Homo sapiens	NA	HuGene-1_0-st	CD43+/41+/235+ progenitors	iPSCs	iPSCs	25621499	8781	6987	4116	1755	2 euploid; 6 trisomy 21	6
GSE62538	GSE62538	NA	Mus musculus	Ts1Cje	MoGene-1_0-st	Ts1Cje.E15	embryonic forebrain (E15)	Forebrain	NA	76	66	48	34	5 euploid; 5 trisomy 21	8
GSE49050.cer	GSE49050	NA	Mus musculus	Ts1Cje	Mouse430_2	Ts1Cje.crblm	Cerebellum	Cerebellum	25052193	174	112	53	38	5 euploid; 5 trisomy 21	8
GSE49050.ctx	GSE49050	NA	Mus musculus	Ts1Cje	Mouse430_2	Ts1Cje.crtx	Cortex	Cortex	25052193	273	149	57	39	5 euploid; 5 trisomy 21	8
GSE49050.hpc	GSE49050	NA	Mus musculus	Ts1Cje	Mouse430_2	Ts1Cje.Hpcmp	Hippocampus	Hippocampus	25052193	438	195	73	47	5 euploid; 5 trisomy 21	8
GSE49635cortex	GSE49635	NA	Mus musculus	Ts1Cje	MoGene-1_0-st	Ts1Cje.crtx	Cortex	Cortex	24916381	92	71	52	37	5 euploid; 6 trisomy 21	9
GSE49635hippocampus	GSE49635	NA	Mus musculus	Ts1Cje	MoGene-1_0-st	Ts1Cje.Hpcmp	Hippocampus	Hippocampus	24916381	136	96	46	25	5 euploid; 6 trisomy 21	9
GSE48611ipsc	GSE48611	NA	Homo sapiens	NA	hgu133plus2	iPSCs	iPSCs	iPSCs	23716668	3646	1975	399	23	3 euploid; 6 trisomy 21	7
GSE48611neurons	GSE48611	NA	Homo sapiens	NA	hgu133plus2	neurons	iPSCs	iPSCs	23716668	81	63	23	8	3 euploid; 6 trisomy 21	7
GSE48051	GSE48051	NA	Homo sapiens	NA	Agilent-014850 Whole Human Genome Microarray 4x44K G4	amniocyte	placenta	Placenta/amnios	24066117	49	21	0	0	9 euploid; 10 trisomy 21	17
GSE42956ipcs	GSE42956	NA	Homo sapiens	NA	Illumina HT-12 v4	iPSCs	fibroblasts	Fibroblasts	23225669	1971	1132	269	57	15 euploid; 12 trisomy 21	25
GSE42956fibroblasts	GSE42956	NA	Homo sapiens	NA	Illumina HT-12 v4	fibroblasts	fibroblasts	Fibroblasts	23225669	571	367	169	26	15 euploid; 6 trisomy 21	19
GSE42772	GSE42772	NA	Homo sapiens	NA	Illumina humanRef-8 v2.0 expression beadchip	astrocytes	astrocytes	astrocytes	23312288	0	0	0	0	3 euploid; 5 trisomy 21	6
GSE42772h2o2	GSE42772	NA	Homo sapiens	NA	Illumina humanRef-8 v2.0 expression beadchip	astrocytes	astrocytes	astrocytes	23312288	0	0	0	0	3 euploid; 5 trisomy 21	6
GSE38931	GSE38931	NA	Homo sapiens	NA	Illumina HumanHT-12 V4.0 expression beadchip	iPSCs	fibroblasts	Fibroblasts	23084023	0	0	0	0	4 euploid; 2 trisomy 21	4
GSE34458	GSE34458	NA	Homo sapiens	NA	Illumina HumanHT-12 V3.0 expression beadchip	lymphoblastoid cell line	Blood	Blood/bone marrow	22912673	135	63	23	0	12 euploid; 11 trisomy 21	21
GSE35665child	GSE35665	NA	Homo sapiens	NA	HuEx-1_0-st	PBMC	Blood	Blood/bone marrow	23155455	134	76	13	3	15 euploid; 10 trisomy 21	23
GSE35665neonate	GSE35665	NA	Homo sapiens	NA	HuEx-1_0-st	PBMC	Blood	Blood/bone marrow	23155455	175	77	14	2	7 euploid; 5 trisomy 21	10
GSE33911	GSE33911	NA	Homo sapiens	NA	Agilent-028004 SurePrint G3 Human GE 8x60K Microarray	iPSCs	iPSCs	iPSCs	NA	1735	81	18	11	3 euploid; 3 trisomy 21	4
GSE23910	GSE23910	NA	Homo sapiens	NA	Agilent-014850 Whole Human Genome Microarray 4x44K G4	thymus	thymus	Thymus	21856934	1	1	0	0	4 euploid; 4 trisomy 21	6
GSE21094	GSE21094	NA	Homo sapiens	NA	hgu133plus2	BM	bone marrow	Blood/bone marrow	21647151	237	52	8	0	26 euploid; 26 trisomy 21	50
GSE24272fetalliver	GSE24272	NA	Mus musculus	Ts1Cje	NimbleGen Mus musculus MM9 Expression Array (12x135k)	fetal liver	fetal liver	Fetal liver	21533146	772	482	229	149	12 euploid; 13 trisomy 21	23
GSE24272placenta	GSE24272	NA	Mus musculus	Ts1Cje	NimbleGen Mus musculus MM9 Expression Array (12x135k)	placenta	placenta	Placenta/amnios	21533146	134	101	57	46	12 euploid; 13 trisomy 21	23
GSE24554	GSE24554	NA	Mus musculus	Ts65Dn	Illumina MouseWG-6 v2.0	Mandible precursor	Mandible precursor	Mandible precursor	NA	0	0	0	0	11 euploid; 13 trisomy 21	22
GSE16677blasts	GSE16677	NA	Homo sapiens	NA	hgu133plus2	AMKL sorted blast	AMKL sorted blast	Blood/bone marrow	20679399	2195	1147	239	12	5 euploid; 6 trisomy 21	9
GSE16677cells	GSE16677	NA	Homo sapiens	NA	hgu133plus2	AMKL cell line	AMKL cell line	Blood/bone marrow	20679399	848	552	272	130	4 euploid; 2 trisomy 21	4
GSE17760	GSE17760	NA	Mus musculus	Ts1Cje	Mouse430_2	neurospheres	lateral walls of the lateral ventricles	NPCs	20661276	2	1	1	1	3 euploid; 3 trisomy 21	4
GSE17459aieop	GSE17459	NA	Homo sapiens	NA	hgu133plus2	white blood cells	Blood	Blood/bone marrow	19965641	7947	5918	2919	1078	72 euploid; 25 trisomy 21	95
GSE17459ich	GSE17459	NA	Homo sapiens	NA	hgu133plus2	white blood cells	Blood	Blood/bone marrow	19965641	1879	1120	427	130	9 euploid; 6 trisomy 21	13
GSE19681	GSE19681	NA	Homo sapiens	NA	hgu133plus2	primary leukemic blasts	Blood	Blood/bone marrow	20194440	0	0	0	0	2 euploid; 3 trisomy 21	3
GSE9805	GSE9805	NA	Mus musculus	Ts1Cje	RNG-MRC_MM25k_EVRY	NPCs	E14 neocortex	Cortex	19472221	0	0	0	0	3 euploid; 3 trisomy 21	4
GSE11448_p0	GSE11448	NA	Mus musculus	Ts1Cje	RNG-MRC_MM25k_EVRY	P0.Cerebellum	Cerebellum	Cerebellum	19331679	21	14	0	0	6 euploid; 6 trisomy 21	10
GSE11448_p3	GSE11448	NA	Mus musculus	Ts1Cje	RNG-MRC_MM25k_EVRY	P3.Cerebellum	Cerebellum	Cerebellum	19331679	0	0	0	0	9 euploid; 8 trisomy 21	15
GSE11448_p7	GSE11448	NA	Mus musculus	Ts1Cje	RNG-MRC_MM25k_EVRY	P7.Cerebellum	Cerebellum	Cerebellum	19331679	2	0	0	0	7 euploid; 8 trisomy 21	13
GSE11448_p10	GSE11448	NA	Mus musculus	Ts1Cje	RNG-MRC_MM25k_EVRY	P10.Cerebellum	Cerebellum	Cerebellum	19331679	96	32	7	0	6 euploid; 6 trisomy 21	10
GSE14021	GSE14021	NA	Mus musculus	TgDyrk1A	Agilent-012694 Whole Mouse Genome G4122A	E12.5.telencephalon	Telencephalon	Cortex	19218269	1	0	0	0	2 euploid; 2 trisomy 21	2
GSE14030	GSE14030	NA	Mus musculus	TgDyrk1A-N18	Agilent-012694 Whole Mouse Genome G4122A	neuroblastoma cell line	neuroblastoma cell line	Neuroblastoma	20457675	1	0	0	0	5 euploid; 5 trisomy 21	8
GSE13123.16_17	GSE13123	NA	Mus musculus	Ts1Cje/Ts65Dn	Illumina MouseWG-6 v1.1	16-17.dpp.sperm cells	sperm cells	Sperm Cells	19639331	2	0	0	0	3 euploid; 3+3 trisomy 21	4
GSE13123.19_20	GSE13123	NA	Mus musculus	Ts1Cje/Ts65Dn	Illumina MouseWG-6 v1.1	19-20.dpp.sperm cells	sperm cells	Sperm Cells	19639331	4	3	1	0	3 euploid; 3+3 trisomy 21	4
GSE11472	GSE11472	NA	Mus musculus	Ts1Cje	Illumina mouse-6 v1.1	cerebellar external granular lay	Cerebellum	Cerebellum	19331679	38	33	20	12	9 euploid; 9 trisomy 21	16
GSE10758	GSE10758	NA	Homo sapiens	NA	Agilent-012097 Human 1A Microarray (V2) G4110B	amniocytes	placenta	Placenta/amnios	18594911	1036	2	0	0	6 euploid; 4 trisomy 21	8
GSE1789	GSE1789	NA	Homo sapiens	NA	hgu133a	fetal heart explant	heart	Heart	17683628	0	0	0	0	3 euploid; 5 trisomy 21	6
GSE4119.amkl	GSE4119	NA	Homo sapiens	NA	hgu133a	AMKL mononuclear cells	Blood	Blood/bone marrow	16492768	3713	2730	1411	552	38 euploid; 23 trisomy 21	59
GSE4119.aml	GSE4119	NA	Homo sapiens	NA	hgu133a	AML mononuclear cells	Blood/bone marrow	Blood/bone marrow	16492768	1470	950	380	82	9 euploid; 2 trisomy 21	9
ETABM473	E-TABM-473	NA	Mus musculus	Tc1	Illumina MouseWG-6 v1.1	hepatocytes	liver	Liver	18787134	30	13	6	0	4 euploid; 4 trisomy 21	6
EMTAB312	E-MTAB-312	NA	Homo sapiens	NA	hgu133a2	EPCs	Endothelial Progenitor Cells	Endothelia	20836844	3906	2422	294	0	4 euploid; 2 trisomy 21	4
EMEXP3355	E-MEXP-3355	NA	Mus musculus	Ts65Dn	MoGene-1_0-st	Heart	Heart	Heart	22693452	85	54	33	1	5 euploid; 5 trisomy 21	8
EMATB1238	E-MTAB-1238	NA	Homo sapiens	NA	hgu133plus2	LCLs	lymphoblastoid cell lines	Blood/bone marrow	23830204	6681	3671	291	2	6 euploid; 6 trisomy 21	10
EMTAB2574_GMP	E-MTAB-2574	NA	Mus musculus	Ts65Dn	Illumina MouseWG-6 v2.0	GMP	Bone marrow	Blood/bone marrow	25973911	2	2	2	2	3 euploid; 3 trisomy 21	4
EMTAB2574_MEP	E-MTAB-2574	NA	Mus musculus	Ts65Dn	Illumina MouseWG-6 v2.0	MEP	Bone marrow	Blood/bone marrow	25973911	3	2	2	2	3 euploid; 3 trisomy 21	4
EMTAB2574_LSK	E-MTAB-2574	NA	Mus musculus	Ts65Dn	Illumina MouseWG-6 v2.0	LSK	Bone marrow	Blood/bone marrow	25973911	15	10	9	7	3 euploid; 3 trisomy 21	4
EMTAB2574_CMP	E-MTAB-2574	NA	Mus musculus	Ts65Dn	Illumina MouseWG-6 v2.0	CMP	Bone marrow	Blood/bone marrow	25973911	0	0	0	0	3 euploid; 3 trisomy 21	4
GSE48553	GSE48553	SRP026566	Mus musculus	Ts1Rhr	Illumina HiSeq 2000	proB-cells cells at passage 1	B-cells	Blood/bone marrow	24747640	108	80	47	20	3 euploid; 3 trisomy 21	4
GSE79842fibroblasts	GSE79842	SRP072769	Homo sapiens	NA	Illumina HiSeq 2500	fibroblasts	ear/chest,arm,leg,thorax, abdmonen,arm	Fibroblasts	27472900	75	50	21	6	6 euploid; 6 trisomy 21	10
GSE79842lymphoblastoid	GSE79842	SRP072769	Homo sapiens	NA	Illumina HiSeq 2500	lymphoblastoid	ear/chest,arm,leg,thorax, abdmonen,arm	Blood/bone marrow	27472900	299	221	104	50	3 euploid; 3 trisomy 21	4
GSE84526	GSE84526	SRP078911	Mus musculus	Dp16	Illumina HiSeq 2500	lineage negative, Sca1 positive	Hematopoietic Stem Cell	Blood/bone marrow	27472900	263	189	116	64	3 euploid; 3 trisomy 21	4
GSE84531monocytes	GSE84531	SRP078912	Homo sapiens	NA	Illumina HiSeq 4000	Monocytes	Monocytes	Blood/bone marrow	27472900	1045	582	258	141	7 euploid; 10 trisomy 21	15
GSE84531tcells	GSE84531	SRP078912	Homo sapiens	NA	Illumina HiSeq 4000	T cells	T cells	Blood/bone marrow	27472900	4484	3060	1220	348	7 euploid; 10 trisomy 21	15
GSE121066	GSE121066	SRP164925	Mus musculus	Ts1Rhr	Illumina NextSeq 500	primary mouse proB_Ts1Rhr c	B-cells	Blood/bone marrow	30428356	3309	2616	1584	817	3 euploid; 3 trisomy 21	4
GSE128621	SRP188973	SRP188973	Homo sapiens	NA	Illumina HiSeq 4000	white blood cells	Blood	Blood/bone marrow	31628327	1100	786	428	241	6+3 euploid; 8+2 trisomic	17
